# Relation between aortic elasticity parameters and SYNTAX score in postmenopausal diabetic women

**DOI:** 10.1186/s43044-023-00358-1

**Published:** 2023-04-25

**Authors:** Mohamed Naseem, Ahmed Alaarag

**Affiliations:** Cardiology Department, Faculty of Medicine, Tanta University, Tanta, Egypt

**Keywords:** Diabetes, Postmenopausal, Aortic elasticity, SYNTAX score

## Abstract

**Background:**

Postmenopausal women are at increased risk of coronary artery disease (CAD). Diabetes Mellitus is a major risk factor for CAD. The stiffening of the aorta is associated with increased cardiovascular morbidity and mortality. We aimed to investigate the relation of aortic elasticity parameters to CAD severity assessed by SYNTAX score (SS) in diabetic postmenopausal women. The study prospectively included 200 consecutive diabetic postmenopausal women with CAD who underwent elective coronary angiography. Patients were classified into 3 groups based on SS, low-SS ≤ 22, intermediate-SS ≥ 23– ≤ 32, and high-SS ≥ 33. Echocardiographic aortic elasticity parameters, including aortic stiffness index (ASI), aortic strain (AS) (%) and aortic distensibility (AD) were obtained in all patients.

**Results:**

Patients in the high SS group were older age and had a higher aortic stiffness. After adjusting different co-variates AD, AS, and ASI could be used as independent predictors of high SS with the following *P*-values (0.019, 0.016 and 0.010) and cut-off values (2.5, 3.6 and 2.9), respectively.

**Conclusions:**

In diabetic postmenopausal women, the simple echocardiography-derived aortic elasticity parameters might predict the severity and complexity of angiographic coronary lesions assessed by the SS.

## Background

Ischemic heart disease is the major cause of morbidity and mortality in diabetic women, and this risk increases in women who have attained menopause [1, 2]. Women have a worse clinical outcome after myocardial infarction and revascularization procedures by stenting or coronary artery bypass grafting [3, 4]. The basis of the increased risk of coronary artery disease (CAD) in postmenopausal women may be related to decreased levels of estrogens [5]. The transition through menopause has been associated with various components of metabolic syndrome, including increased central body fat, dyslipidemia, increased glucose levels, and insulin resistance [6]. Women with diabetes mellitus (DM) have a higher cardiovascular (CV) risk relative to men [7]. Arterial stiffness may be an important underlying pathophysiological mechanism linking diabetes to increased CV risk [8]. Loss of arterial elasticity is associated with worse CV outcomes [9]. The aortic stiffness index (ASI) and Aortic distensibility (AD) are two measures of aortic elasticity, both are associated with CAD fatal and non-fatal events [10, 11]. Arterial stiffness can be assessed by various non-invasive methods such as applanation tonometry, echocardiography, and magnetic resonance imaging [12]. Echocardiography is the most widely used imaging technique in clinical CV practice [13]. Echocardiography‑derived indices, including simple M-mode-derived measurements, may be more reliable, as central arterial elasticity is more important than peripheral arterial elasticity in predicting the CV outcome [14]. Some studies reported an association between impaired indices of aortic elasticity and the severity of CAD [15]. The current study aims to assess the relation of aortic elasticity parameters to CAD severity assessed by SYNTAX score (SS) in diabetic postmenopausal women.

## Methods

### Study population

The study prospectively included 200 consecutive postmenopausal women with type 2 DM presenting to Tanta University, Cardiology department during the period from September 2022 till December 2022 and were referred for elective coronary angiography for diagnosis of CAD based on patients’ symptoms, positive stress test, and electrocardiographic evidence of ischemia.

Informed consent was taken from all patients, and the study was approved by the local ethical committee.

The diagnosis of diabetes was based on the clinical history of pre-existing DM (indicated by insulin or oral antidiabetic medication use), fasting plasma glucose ≥ 126 mg/dL, 2-h plasma glucose ≥ 200, a random plasma glucose ≥ 200 mg/dL or HbA1c ≥ 6.5% any time [16].

Menopause is defined as women with the absence of a menstrual period for at least 12 consecutive months and not using a hormonal contraceptive [17].

Exclusion criteria were: Poor echogenic window, patients presenting with acute coronary syndrome, left ventricular ejection fraction (LVEF) < 50%, use of hormone replacement therapy, those without significant CAD (luminal stenosis < 50%), normal coronary angiography, patients on hemodialysis, collagen vascular diseases, congenital heart disease, more than mild valvular stenosis or regurgitation, prior percutaneous coronary intervention, history of coronary artery bypass surgery, prosthetic heart valves, atrial fibrillation, atrial flutter.

**Fig. 1:**
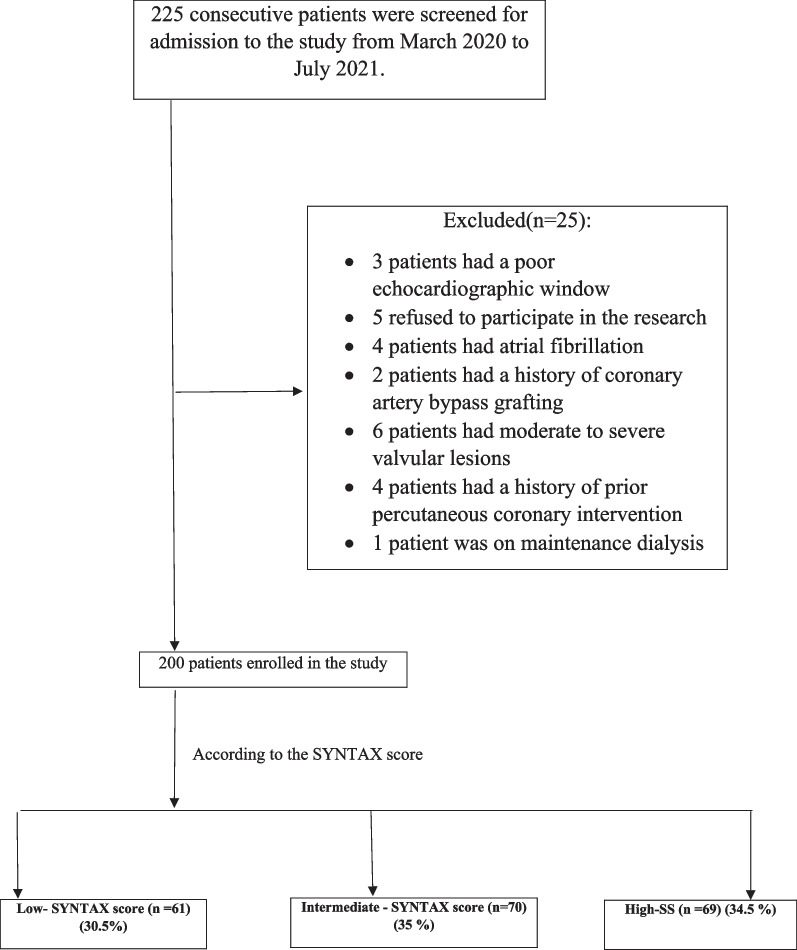
Flowchart of the patients included in the study

As the flowchart shows in Fig. 1, during the enrolment period, 225 consecutive patients were screened for admission to the study. For various reasons, 25 were not considered eligible: 3 patients had a poor echocardiographic window, 5 refused to participate in the research, 4 patients had atrial fibrillation, 2 patients had a history of coronary artery bypass grafting, 6 patients had moderate to severe valvular lesions, 4 patients had a history of prior percutaneous coronary intervention (PCI), and 1 patient was on maintenance dialysis.

Blood pressure (BP) measurements were obtained just before starting the echocardiographic study. Blood pressure values were obtained in the sitting position after at least 5 min of rest in a quiet room. BP was measured three times with mercury sphygmomanometers at 1–2 min intervals. Systolic and diastolic BP was recorded as the average of the last two BP measurements, and the difference between them was defined as pulse pressure [18].

### Angiographic procedure

Coronary angiography was performed using the standard techniques through the femoral or radial approach. The SS was computed from the diagnostic coronary angiogram as the sum of the points for each significant coronary lesion (defined as diameter stenosis > 50% in vessels with minimum diameter > 1.5 mm). Patients were divided according to the SS into three groups: Low‑SS ≤ 22, intermediate‑SS ≥ 23– ≤ 32, and high‑SS ≥ 33 [19]

### Echocardiographic evaluation

Two dimensional transthoracic echocardiographic and Doppler studies were performed using the commercially available GE Vivid 7 echocardiograph with a 2.5 MHz transducer. LVEF was evaluated using the modified Simpson formula [20]

In the parasternal long-axis view the systolic and diastolic inner diameters of the ascending aorta were measured by M-mode tracing 3 cm distal to the aortic valve.

The aortic systolic diameter (AoS) was measured at the maximal anterior motion of the aortic valve, whereas the diastolic diameter (AoD) was measured at the peak of the QRS complex on the simultaneously recorded electrocardiogram. The measurements were averaged over 3 consecutive cardiac cycles. Aortic elasticity indices were calculated according to these formulas:

$$\begin{aligned} & {\text{ASI}} = {\text{ In}}\;\left( {{\text{SBP}}/{\text{DBP}}} \right)\;\left[ {\left( {{\text{AoS}} - {\text{AoD}}} \right)/{\text{AoD}}} \right]\;\;\left[ {{21}} \right] \\ & {\text{Aortic}}\;{\text{strain}}\;\left( {{\text{AS}}} \right)\;\left( \% \right) = {1}00 \times \left( {{\text{AoS}} - {\text{AoD}}} \right)/{\text{AoD}}\;\;\left[ {{22}} \right] \\ & {\text{AD}}\;\left[ {{1}/\left( {{1}0{3} \times {\text{mmHg}}} \right)} \right] = {2} \times \left[ {\left( {{\text{AoS}} - {\text{AoD}}} \right)/{\text{AoD}}} \right]/{\text{PP}}\;\;\left[ {{21}} \right] \\ & {\text{Stroke}}\;{\text{volume}}\;\left( {{\text{SV}}} \right)\;\left( {{\text{mL}}} \right) = {\text{LV}}\;{\text{outflow}}\;{\text{tract}}\;{\text{area}} \times \left( {{\text{LV}}\;{\text{outflow}}\;{\text{tract}}\;{\text{time}} - {\text{velocity}}\;{\text{integral}}} \right) \;\;\left[ {{23}} \right] \\ & {\text{Stroke}}\;{\text{volume}}\;{\text{index}}\;\left( {{\text{SVi}}} \right)\;\left( {{\text{mL}}/{\text{m}}^{{2}} } \right) = {\text{SV}}/{\text{BSA}}\;\;\left[ {{23}} \right] \\ \end{aligned}$$

### Reproducibility

n experienced echocardiographer performed all measurements. In randomly selected 15 patients intra-observer and inter-observer variability of aortic elasticity indices were evaluated using intraclass correlation coefficients by repeated analysis by the same investigator or independently by two separate investigators.

### Statistical analysis

Statistical analyses were performed using the IBM SPSS software package version 20.0. (Armonk, NY: IBM Corp). The Kolmogorov- Smirnov test was used to test for the normal distribution of continuous data. The obtained quantitative data were presented as mean, standard deviation (SD), and qualitative data were expressed as numbers and percentages. Results were analyzed using one-way analysis of variance (ANOVA) when comparing between more than two means, and the Post Hoc test was used for multiple comparisons between different groups. A Chi-square (X2) test of significance was used in order to compare proportions between two qualitative parameters.

Univariate and multivariable logistic regression analyses were performed to detect potential independent predictors of high SS. Receiving operator characteristics (ROC) curve was used to detect optimal cut-off values of aortic elasticity parameters for predicting a high SS. A *P* value < 0.05 is considered as statistically significant.

In addition, the power of the sample size was calculated by G Power tool (Franz Faul, University of Kiel, Germany, version 3.1.9.4) with 0.05 alpha and 0.25 effect size. The calculated power value was 0.89 according to post hoc-type power analysis.

## Results

This cross-section study included two hundred postmenopausal female patients who attended the cardiology department for elective coronary angiography.

Patients were classified into three groups according to the severity of coronary artery disease as measured by the SS: low, intermediate, and high SS groups.

The clinical, hemodynamic, laboratory and echocardiographic parameters of the aortic stiffness of the three groups were analyzed (Table 1). There is no statistically significant difference between the three groups regarding the incidence of smoking, hypertension, body mass index (BMI), duration of diabetes diagnosis, age at menopause, hemoglobin A1c (HBA1c) level, lipid profile, LVEF, heart rate, aortic diameter in both systole and diastole, stroke volume, stroke volume index, and pulse pressure/stroke volume index ratio.

**Table 1:** Demographic, clinical characteristics, laboratory, and Echocardiographic parameters of the study groups

		Low SS (n = 61)	Intermediate SS (n = 70)	High SS (n = 69)	F/ X^2^	*P*. value	P1	*P*2	*P*3
Age	Mean ± SD	54.21 ± 5.00	56.37 ± 5.78	60.38 ± 5.59	21.351	0.001*	0.026*	0.001*	0.001*
Smoking	N	2	1	2	0.526	0.769	0.480	0.900	0.551
	%	3.3%	1.4%	2.9%					
Hypertension	N	19	21	19	0.216	0.898	0.887	0.651	0.748
	%	31.1%	30.0%	27.5%					
BMI (Kg/m2)	Mean ± SD	30.33 ± 3.65	29.52 ± 3.97	30.06 ± 3.45	0.831	0.437	0.211	0.677	0.389
*Medications*									
ACEI	N	36	44	39	0.587	0.746	0.653	0.774	0.446
	%	60%	63%	56%					
BB	N	26	31	33	0.381	0.828	0.848	0.552	0.675
	%	42%	44%	48%					
CCB	N	10	14	13	0.287	0.865	0.595	0.715	0.863
	%	16%	20%	19%					
Statins	N	36	43	40	0.182	0.914	0.778	0.904	0.678
	%	59%	61%	58%					
*Anti-diabetic treatment*									
Insulin	N	20	21	25	0.612	0.736	0.732	0.680	0.435
	%	32%	30%	36%					
Oral hypoglycaemics	N	35	41	39	0.063	0.970	0.890	0.922	0.807
	%	57%	58%	56%					
Insulin + oral hypoglycaemics	N	6	8	5	0.719	0.698	0.769	0.596	0.397
	%	10%	11%	7%					
Time since diabetes diagnosis (Years)	Mean ± SD	5.54 ± 2.64	5.50 ± 2.63	5.68 ± 2.70	0.088	0.916	0.930	0.764	0.688
Age at menopause (Year)	Mean ± SD	47.62 ± 1.53	47.86 ± 1.52	47.90 ± 1.43	0.633	0.532	0.371	0.294	0.870
HBA1c (%)	Mean ± SD	7.02 ± 0.62	7.01 ± 0.62	7.01 ± 0.55	0.002	0.998	0.971	0.951	0.979
LDL (mg/dl)	Mean ± SD	130.36 ± 42.28	137.62 ± 37.63	127.90 ± 38.21	1.143	0.321	0.293	0.722	0.147
HDL (mg/dl)	Mean ± SD	44.56 ± 8.05	45.51 ± 7.40	44.23 ± 7.73	0.516	0.597	0.480	0.811	0.328
TG (mg/dl)	Mean ± SD	208.75 ± 47.15	214.07 ± 44.16	206.39 ± 44.79	0.523	0.594	0.504	0.767	0.319
LVEF %	Mean ± SD	60.79 ± 5.00	60.71 ± 5.12	60.90 ± 4.92	0.024	0.977	0.934	0.899	0.829
Heart Rate (B/min)	Mean ± SD	66.95 ± 6.62	65.87 ± 6.32	66.09 ± 6.37	0.504	0.605	0.339	0.445	0.844
SBP (mmHg)	Mean ± SD	113.52 ± 12.92	105.36 ± 8.40	106.01 ± 10.38	11.625	0.001*	0.001*	0.001*	0.716
DBP (mmHg)	Mean ± SD	83.11 ± 9.92	74.71 ± 7.01	72.46 ± 8.34	28.190	0.001*	0.001*	0.001*	0.118
Pulse pressure	Mean ± SD	30.41 ± 6.08	30.64 ± 5.24	33.55 ± 5.82	6.340	0.002*	0.816	0.002*	0.003*
AoS(cm)	Mean ± SD	3.40 ± 0.49	3.38 ± 0.49	3.40 ± 0.48	0.080	0.923	0.733	0.995	0.730
AoD (cm)	Mean ± SD	3.19 ± 0.51	3.18 ± 0.51	3.30 ± 0.48	1.191	0.306	0.868	0.228	0.156
Aortic distensibility(cm^2^/dyn/103)	Mean ± SD	4.83 ± 2.19	4.32 ± 1.84	2.01 ± 0.67	53.748	0.001*	0.079	0.001*	0.001*
Aortic strain %	Mean ± SD	6.99 ± 2.86	6.51 ± 2.73	3.27 ± 0.86	51.279	0.001*	0.239	0.001*	0.001*
Aortic stiffness index	Mean ± SD	2.449 ± 0.470	2.482 ± 0.463	3.062 ± 0.230	50.036	0.001*	0.641	0.001*	0.001*
SV (ml)	Mean ± SD	68.85 ± 8.91	69.27 ± 7.80	68.59 ± 8.16	0.118	0.888	0.773	0.859	0.630
SVi (ml/m^2^)	Mean ± SD	44.06 ± 7.46	43.74 ± 4.19	43.78 ± 3.75	0.069	0.933	0.731	0.764	0.965
PP/SVi	Mean ± SD	0.81 ± 0.97	0.71 ± 0.16	0.77 ± 0.14	0.601	0.549	0.279	0.652	0.514

P1, significance between Low & Intermediate SS; P2, significance between Low & High SS; P3, significance between Intermediate & High SS; SS, YNTAX score; HBA1c, Haemoglobin A1c; LDL, Low-Density Lipoprotein; HDL, Low-Density Lipoprotein; TG, Triglycerides BMI, Body Mass Index; ACEI, Angiotensin-converting enzyme inhibitors; BB, Beta-blockers; CCB, Calcium channel blockers; B/min (beat/ minute); LVEF, Left Ventricular Ejection Fraction; SBP, Systolic Blood Pressure; DBP, Diastolic Blood Pressure; AO, Aorta;SV, Stroke Volume; SVI, Stroke Volume Index; PP, Pulse pressure

*Significant *P*-value

On the other hand, there was a statistically significant difference between the three groups regarding age, systolic blood pressure, diastolic blood pressure, pulse pressure, AD, AS, and ASI with *P*-values of (0.001, 0.001, 0.001, 0.002, 0.001, 0.001, and 0.001), respectively (Table 1). Furthermore, the post hoc test was performed, which showed that patients with a high SS had a statistically significant higher pulse pressure, ASI, and AD with lower AS than patients with low and intermediate SS with *P*-values for pulse pressure of (0.002 and 0.003) respectively, ASI (0.001 and 0.001) respectively and AD (0.001 and 0.001) respectively and AS (0.001 and 0.001) respectively. However, there was no statistically significant difference regarding these parameters in low and intermediate SS groups.

In the same context, the post hoc test showed that there is a statistically significant increase in age with the increase in SS with a statistically significant difference between low and intermediate, low and high as well as intermediate and high score groups with *P*-values of (0.026, 0.001 and 0.001) respectively.

There were statistically significant differences between patients with high and low SS as well as patients with intermediate and high SS regarding the systolic and diastolic blood pressure at enrollment with *P*-values of 0.001 for both.

Univariate and multivariable logistic regression models were built to identify potential predictors of high SS. The results showed that age, AD, AS, and ASI are independent predictors for high SS with *P*-values of (0.027, 0.019, 0.016, and 0.010) respectively (Table 2).

**Table 2:** Univariate and multivariate analysis of predictors of high SYNTAX score

	Univariate		Multivariate	
	OR (95% CI)	*P* value	OR (95% CI)	*P* value
Age	0.684 (0.451–0.796)	0.003*	0.237(0.174–0.594)	0.027*
SBP	2.634 (1.574–4.872)	0.016*	1.854 (0.834–3.527)	0.103
DBP	1.857 (1.216–2.364)	0.008*	1.324 (0.635–2.417)	0.091
Pulse pressure	0.617 (0.234–0.761)	0.007*	0.847 (0.327–1.851)	0.164
Aortic distensibility	1.954 (1.108–3.627)	0.001*	1.236 (1.017–2.864)	0.019*
Aortic strain	2.415 (1.864–3.627)	0.001*	1.964 (1.306–2.875)	0.016*
Aortic stiffness index	0.527 (0.234–0.867)	0.001*	0.765 (0.507–0.913)	0.010*

SBP, systolic blood pressure; DBP, diastolic blood pressure

*Significant *P*-value

**Fig. 2:**
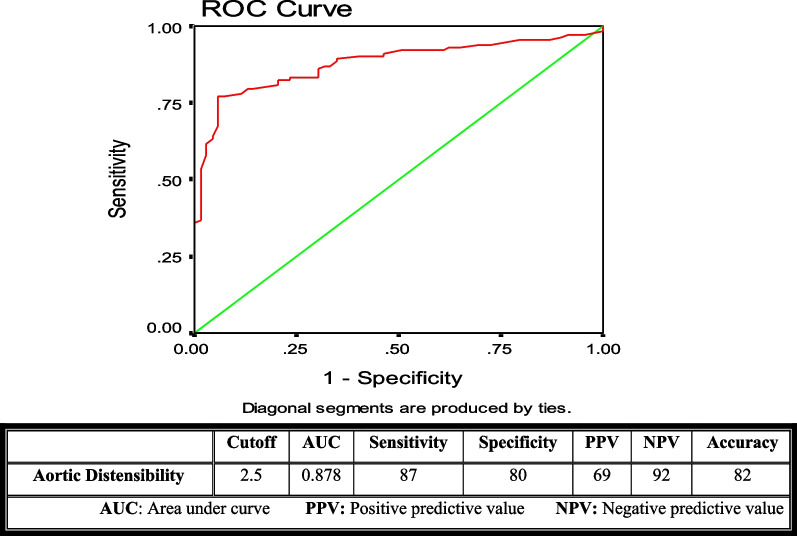
The receiving operator characteristics curve for aortic distensibility

**Fig. 3:**
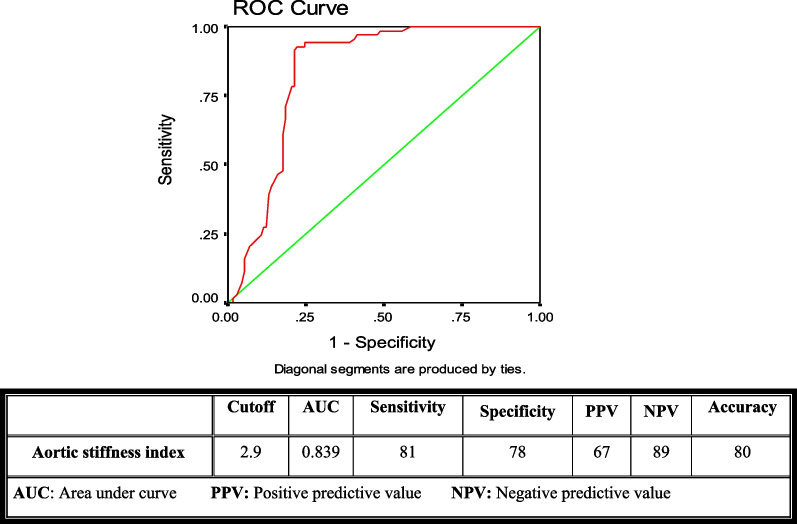
The receiving operator characteristics curve for aortic stiffness index

**Fig. 4:**
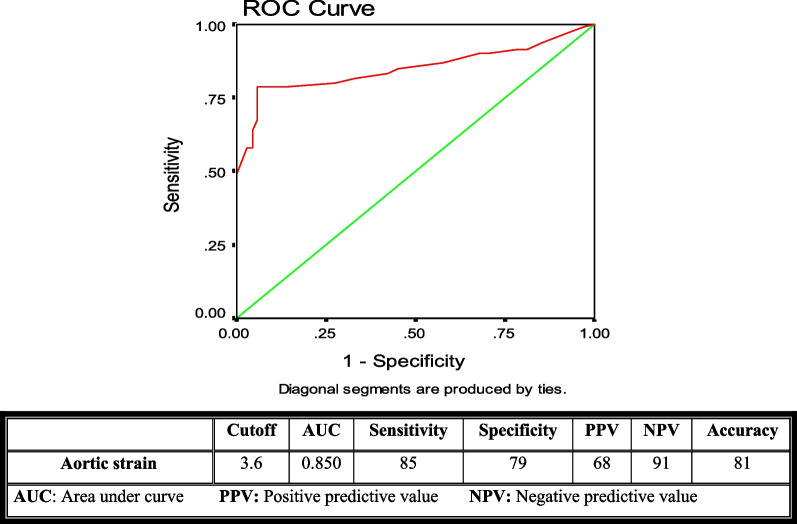
The receiving operator characteristics curve for aortic strain

In the ROC curve analysis of the above-mentioned aortic elasticity parameters, the best cut-off values for AD, ASI, and AS were: (2.5, 2.9, and 3.6) with sensitivity (87, 81, and 85), specificity (80, 78. 79) and the area under the curve of (0.878, 0.839 and 0.850) respectively (Figs. 2, 3, and 4).

### Reproducibility

Intra-observer and inter-observer variability for aortic elasticity indices measurements ranged from 0.93 and 0.96.

## Discussion

Menopause is associated with an increased incidence, progression, and severity of metabolic syndrome [24]; Large systemic studies have shown a relationship between age and arterial stiffness; however, these studies did not examine the prevalence of menopause or assess the relationship in a specific age range [25]

Smulyan et al. observed in their study that structural alterations of the blood vessel caused age-related variations in pulse wave velocity in women; however, they did not expressly take into account the influence of menopause in their study. Palmiero et al. [26] in their study, showed that postmenopausal women have increased aortic stiffness compared to controls [27].

Also, after adjusting for different cardiovascular risk factors in the SWAN study, arterial stiffness was found to be increased by 7.5% within one year of the final menstrual period; they used the carotid-femoral pulse wave velocity as a marker for aortic stiffness [28].

On contrary to these findings and to the findings in our study, the cross-sectional study by, Lodon et al. showed that menopause reduces the age-related rise in arterial stiffness, although their sample size was too small [29].

Saltiki et al. [2]. examined the effect of diabetes mellitus on CAD severity in postmenopausal women; they reported that diabetic postmenopausal women had more severe CAD compared to non-diabetic women, as evidenced by greater diseased vessels with more than 50% stenosis when compared with non-diabetic women. Similarly, Mellen et al. [30] found that diabetes mellitus was associated with angiographic progression of CAD and poor CV outcome in postmenopausal women.

In a retrospective analysis of the Women's Angiographic Vitamin and Estrogen (WAVE) trial Ahmad et al. [31] discovered a complex relationship between DM and the evolution of CAD in postmenopausal women they found even at low HbA1c rates, clinically evident DM, rather than increased glycosylated hemoglobin (HbA1c) alone, appears to enhance the advancement of established coronary lesions.

After menopause, both the prevalence and mortality of CAD in women rise. Age, abdominal obesity, and in particular (DM), combined with ovarian function loss and the resulting lack of endogenous estrogens, accelerate the development of atherosclerosis. It is generally known that estrogen positively affects some of the main CAD risk factors [32].

The hallmark of the current study is that the increased aortic stiffness as measured by AD, ASI, and the AS was related to more aggressive coronary atherosclerosis and high SS in diabetic postmenopausal women. In our cohort, this finding was not dependent on other traditional cardiometabolic risk factors such as smoking status, history of hypertension, BMI, HBA1c, and lipid profile levels as these factors did not show any significant difference between the studied groups.

Moreover, in the multivariate analysis, after adjusting different variables that can affect the severity of coronary artery disease, the parameters mentioned above were found to be independent predictors of a high SS.

Aortic elasticity plays a vital role in maintaining blood flow during diastole. It regulates the pulsatile flow of blood from the heart and makes a steady state of flow to different body organs [33]. Arterial stiffness leads to end-organ damage [34, 35] and can lead to reduced coronary blood flow [36] and left ventricular hypertrophy [37].

It is difficult to ignore the impact of age when analyzing how menopause affects arterial stiffness. However, it has been documented that estrogen deficit in postmenopausal women worsens the effects of aging on arterial stiffness. Additionally, the renin-angiotensin system is also activated, atherogenic inflammatory cytokines are produced, and collagenase activity is decreased by estrogen deficiency [25].

Estrogen receptors α and β are present in the human vasculature, and estradiol is thought to keep the elasticity of the aorta through vasodilatation and vascular matrix formation [28]. There is strong evidence that apart from the classic cardiovascular risk factors, the mechanism of aortic stiffness involves a process of inflammation and cytokines activation [38], and menopause is associated with a state of low-grade systemic inflammation that may help in the progression of aortic stiffness [39].

Many studies used the carotid-femoral pulse-wave velocity [28] and ankle-brachial index [40] as a marker of arterial stiffness. They proved that aortic stiffness could predict cardiovascular risk, especially in postmenopausal women.

Similar to our study Karakurt et al. [41] found that ASI, AS, and elasticity were related to the severity of coronary artery disease; however, their study included three hundred sixty-seven patients of both sex. In the same context, El-Naggar et al. [42] concluded in their study that simple M-mode-derived aortic elasticity indices might predict patients with more severe and complex CAD. All these data are matched with our findings.

The limitation of this study includes the small sample size as we calculated the sample size to the whole study population for each group; we can not exclude the influence of age on our results.

## Conclusions

In diabetic postmenopausal aortic elasticity parameters evaluated by echocardiography might predict high SS.

## Data Availability

The datasets used and or analyzed during the current study are available from the corresponding author upon request.

## Abbreviations

CAD: Coronary artery disease
CV: Cardiovascular risk
SS: SYNTAX score
LVEF: Left ventricular ejection fraction
PCI: Percutaneous coronary intervention
AoS: Aortic systolic diameter
AoD: Diastolic diameter
ASI: Aortic stiffness index
AS: Aortic strain
AD: Aortic distensibility
SV: Stroke volume
Svi: Stroke volume index
BMI: Body mass index
HbA1c: Hemoglobin A1c
